# USPHS and IDSA collaborate on guidelines to prevent opportunistic infections in HIV-infected persons.

**DOI:** 10.3201/eid0103.950314

**Published:** 1995

**Authors:** J E Kaplan, K K Holmes


### Japanese Encephalitis Acquired in Australia

Japanese encephalitis (JE), a mosquito-borne flaviviral disease of humans and animals, is a major public health problem in Asia, where an estimated 50,000 cases occur each year. There has been concern that the range of epidemic JE may be expanding.

On April 5, 1995, an outbreak of three cases of JE was recognized in Australia. Two of the cases were fatal; all were among residents of an island in Australia's Torres Strait, which lies between mainland Queensland and Papua New Guinea. JE was confirmed in two of the patients by polymerase chain reaction (Jeffrey Hanna, Queensland Health, pers. comm.). No other cases were reported. This is the first recognized episode of JE acquired in Australia.

Control activities on the Australian island began on April 7. The community was informed about the importance of personal mosquito protection measures. In addition, larvicides were applied, and areas were fogged to kill adult mosquitoes.

The patients were all male, aged 6 to 44 years. All were hospitalized with symptoms that included fever (up to 40ºC), stiff or painful neck, headache, and abdominal pain. Two patients were unconscious at the time of admission.

Acute-phase sera showed elevated JE virus immunoglobulin M (IgM) titers. Two of the patients also had detectable levels of Kunjin and Murray Valley encephalitis virus IgM, but the JE IgM titers were significantly higher in each case.

Flaviviruses have also been isolated from the sera of each of two asymptomatic island residents. Preliminary tests suggest that these are both JE virus. Blood taken from 10 horses and 12 domestic pigs living near humans on the island was also tested. All 12 pigs and 9 of the horses had high JE titers by hemagglutination inhibition assay. Neutralizing antibody to JE virus was detectable in all the pigs and in four of the horses tested to date.

Details of the index case are as follows: The patient, a 16-year-old male, was admitted to Thursday Island Hospital on March 22, 1995. He was unconscious and was responsive only to painful stimuli. His neck was stiff, and he showed a preference for moving his right side. His illness had begun 3 days before. The day before admission he complained of abdominal pain. This patient had been mildly mentally retarded since birth and occasionally had generalized seizures but was generally healthy. He was transferred to Cairns Base Hospital, where a cerebral CT scan showed a nonenhancing hypodense lesion in his posterior right basal ganglia.

He had a leukocytosis of $17.3 \times 10^9$L, neutrophils, $15.2 \times 10^9$. His cerebrospinal fluid contained 150 leukocytes/µl with a differential count of 50% polymorphs and 50% mononuclear cells.

He had a generalized seizure and 2 days after admission, required mechanical ventilation. He never regained consciousness and died on day 17 of hospitalization (April 8).

---

Adapted from Hanna J, Ritchie S, Loewenthal M, et al. Probable Japanese encephalitis acquired in the Torres Strait. Communicable Diseases Intelligence 1995;19:206-7.

### USPHS and IDSA Collaborate on Guidelines to Prevent Opportunistic Infections in HIV-Infected Persons

U.S. Public Health Service (USPHS)/Infectious Diseases Society of America (IDSA) Guidelines for Preventing Opportunistic Infections in HIV-Infected Persons will be published in an August 1995 supplement of *Clinical Infectious Diseases*. The guidelines, which are intended for health care providers, are the result of collaboration between the Centers for Disease Control and Prevention (CDC), the National Institutes of Health, IDSA, numerous federal and nonfederal organizations, community groups, and HIV-infected persons. The guidelines are endorsed by the American Academy of Pediatrics, the Infectious Diseases Society of Obstetrics and Gynecology, and the Society of Healthcare Epidemiologists of America. Jonathan E. Kaplan, M.D. (CDC), Henry Masur, M.D. (NIH), and King Holmes, M.D., Ph.D. (University of Washington), chaired the USPHS/IDSA Prevention of Opportunistic Infections Working Group and are guest editors of the *Clinical Infectious Diseases* supplement.

CDC initiated work on the guidelines in early 1994; meetings were held in Atlanta in June and September to discuss and refine the recommendations.

The USPHS/IDSA guidelines address 17 opportunistic infections from three angles: 1) preventing exposure to opportunistic pathogens (e.g., sexual, occupational, and environmental exposure as well as exposure through pets, food, water, and international travel); 2) preventing opportunistic disease by chemoprophylaxis and vaccination; and 3) preventing disease recurrence. In this document, new recommendations were made and earlier recommendations were updated. For example, new guidelines recommend that in nonemergency situations, cytomegalovirus (CMV)-seronegative HIV-infected persons who require blood transfusions receive only

CMV-antibody-negative or leukocyte-reduced cellular blood products. The guidelines also recommend that *Toxoplasma*-seropositive HIV-infected persons who have a CD4+ lymphocyte count <100 cells/µL received chemoprophylaxis against toxoplasmosis (such chemoprophylaxis is generally accomplished with anti-*Pneumocystis carinii* medication). Earlier recommendations for chemoprophylaxis against *Pneumocystis carinii* pneumonia and *Mycobacterium avium* complex disease have also been updated.

In addition to disease-specific recommendations, the guidelines include an overview article designed to prioritize the recommendations for health care providers. This article provides an approach to the initial and follow-up evaluations of the HIV-infected patient and also contains sections on HIV-infected pregnant women and HIV-exposed/infected children. The guidelines are followed by 15 background articles, which provide the information on which the recommendations were based and include research priorities generated by the development of the prevention recommendations.

The guidelines conclude with quality standards and implementation steps on the most standard-of-care recommendations, such as chemoprophylaxis against *Pneumocystis carinii* pneumonia. This final section provides a mechanism by which health care facilities can assess their degree of compliance with the recommendations, so that they can detect and correct compliance-related problems.

An abbreviated version of the USPHS/IDSA Guidelines will be published in CDC's *Morbidity and Mortality Weekly Report* in July.

**Jonathan E. Kaplan**
Centers for Disease Control and Prevention
Atlanta, Georgia, USA

**Henry Masur**
National Institutes of Health
Bethesda, Maryland, USA

**King K. Holmes**
University of Washington
Seattle, Washington, USA

### Recommendations for a Regional Strategy for the Prevention and Control of Emerging Infectious Diseases in the Americas

On June 14-15, 1995, a conference on "Combating Emerging Infectious diseases: Challenges for the Americas" was held at the Pan American Health Organization (PAHO) Headquarters in Washington, D.C. The meeting was designed to shape a regional strategy for preventing and controlling emerging infectious diseases that could pose serious threats to the peoples of the Americas.

Participants, convened by PAHO, included top officials and infectious disease experts from that organization as well as the World Health Organization, the U.S. Centers for Disease Control and Prevention, the Canadian Laboratory Center for Disease Control, the U.S. Department of Defense, and several Latin American and Caribbean countries.

This international group of experts noted that an increasing number of new, emerging, and reemerging infectious diseases have been identified in both developed and developing nations and that these diseases threaten to increase in the near future. They include human immunodeficiency virus/acquired immunodeficiency syndrome, which emerged in the l980s and now affects some 16 million people worldwide; and cholera, which returned to the Western Hemisphere for the first time this century in 1991 and has caused more than 1 million cases and 9,000 deaths in the Americas. PAHO estimates that it will take more than a decade and over $200 billion to control the current pandemic of this disease.

The experts concluded that both early warnings of, and rapid responses to, infectious disease threats are needed. The group made several major recommendations to PAHO and its member states to improve surveillance, research, and communications in developing countries. They also issued more detailed recommendations in the areas of antimicrobial resistance, outbreak control, and information and communication. In addition, a plan of action is forthcoming.

The group made the following recommendations for PAHO and its member countries:

### General Recommendations

- Develop and frequently update prioritized disease-specific guidelines for the prevention and control of diseases that are emerging or reemerging, both at the public health and individual levels. This should include biologic and behavioral change measures and will require groups of experts for each disease as well as communications experts. Diseases of interest include yellow fever, dengue, antimicrobial-resistant organisms (malaria, tuberculosis, and enteric diseases), measles, polio, cholera and other foodborne and waterborne diseases, viral hemorrhagic fevers, plague, rabies and other zoonoses, and trypanosomiasis and other vector-borne diseases.
- Identify points of contact in the field to receive and transmit information in countries. These contacts should include organizations and individuals outside the government.

